# Functional MRI study of language organization in left-handed and right-handed trilingual subjects

**DOI:** 10.1038/s41598-020-70167-y

**Published:** 2020-08-05

**Authors:** Sandrine Yazbek, Tarek Smayra, Iyad Mallak, Stephanie Hage, Ghassan Sleilaty, Chirine Atat, Joe Abdel Hay, Ronald Moussa

**Affiliations:** Hotel Dieu de France Hospital, Boulevard Alfred Naccache, Achrafieh, PO Box: 166830, Beirut, Lebanon

**Keywords:** Neuroscience, Medical research, Neurology

## Abstract

Functional MRI (fMRI) is gaining importance in the preoperative assessment of language. Selecting the appropriate language to test by fMRI in trilingual patients is intricate. Our objective is to compare fMRI maps for all three languages in left- and right-handed trilingual subjects. 15 right- and 15 left-handed trilingual volunteers were included in the study. We performed fMRI for each volunteer with a visual responsive naming paradigm that was repeated three times, once in each language. The activated areas and the laterality indices were calculated and correlation with the age of acquisition and proficiency of each language was determined. Strong statistical correlation was found between the Laterality Index (LI) of the three languages, in both the right and left-handed groups. Discordant lateralization of language was only observed in four left-handed subjects who demonstrated bilateral and left-lateralization. In right-handed subjects, the activation maps for the first and the second acquired language were similar. The largest activation was seen with the last acquired language. Irrespective of language proficiency and age of acquisition, the language lateralization might change for left-handed subjects. In right-handed subjects, there is no change and the last acquired language results in the largest activation. fMRI performed for a single language can accurately determine language lateralization in right-handed subjects, whereas in left-handed subjects, it is mandatory to test all languages.

## Introduction

The number of languages spoken throughout the world is reported to be around 6,000^1^, 30 times the number of countries in the world^2^. At least more than half the world’s population is bilingual^3^. Extensive research has been conducted on language organization in bilingual^4–11^ and multilingual populations^12–16^. Several factors have been identified as playing a role in the organization of language in multilingual subjects. They include: the age of second language (L2) acquisition^5,17^, the proficiency level^18^, the length of use and exposure to a language^19^, and the linguistic differences between L1 (the first language acquired) and L2 (the second language acquired)^20^. Bilinguals are divided into early or simultaneous bilinguals with an Age of Acquisition (AoA) less than 6 years of age and late or sequential bilinguals with an AoA after 6 years of age. Early multilinguals have been reported to have recruitment of additional brain areas, including regions in the right hemisphere which leads to a more bilateral organization of their languages compared to monolinguals and late bilinguals^5,21^. On the other hand, late multilinguals present greater activation compared to early multilinguals^18^.

Proficiency also plays a role in language organization. Activations associated with the less proficient language in bilinguals are more significant and widespread^18,22^ and may involve additional areas such as supplementary motor cortex during reading for instance^23^. Less proficient, late bilingual speakers tend to have more widespread activations in their L2 due to recruitment of areas associated with increased cognitive effort^5,12,24^. High proficient multilingual speakers have more sites that are positive for L1 than L2 or L3 on intraoperative language mapping^13,25,26^.

Functional MRI (fMRI) is now routinely used for language assessment and localization in the preoperative neurosurgical setting^27,28^. It has replaced the intracarotid sodium amobarbital procedure (Wada test^29^), as it is a less invasive modality that carries fewer complications, does not involve radiation, and gives similar results regarding language dominance. In addition, it helps determine the organization of the main and accessory language areas in the brain, and their anatomic localization and distance from a brain lesion^30,31^. There is also a good agreement between fMRI and intraoperative electrocorticography^32^. By helping to decide whether a lesion can be resected without compromising language, fMRI plays a central role in the surgical planning. It also abates the time involved for awake cortical mapping thus reducing the surgical time^32,33^. With globalization, more access to internet and large-scale migrations of population, physicians are expected to treat and operate on bilingual, if not multilingual patients, on a frequent basis. Defining which language to test by fMRI becomes of essence in multilingual patients.

Prior research has demonstrated that there is a good concordance between language Laterality Index (LI) values in bi- and multilinguals on fMRI^4,5^. However, studies on multilingual individuals were mainly centered on right-handed subjects. The number of the left-handed individuals that were included in the above-mentioned studies was small, 2 out of 16 subjects were left-handed in the study by Centeno et al.^4^, and three left-handers out of 25 subjects were included in the paper by Polcynska et al.^11^. The left-handed individuals constitute a different population than the right-handed population in terms of language lateralization. The left-handers present more frequent atypical language lateralization than the normal right-handed subjects (22% vs 4–6%)^34^ and right hemisphere participation is frequent in normal left-handed subjects^35^. Evaluation of language organization in multilingual left-handed subjects remains under reported and under investigated. The objective of our study is to assess language organization in left-handed healthy multilingual individuals and to establish whether testing one single language reliably determines language lateralization and localization in trilingual left-handed and right-handed subjects. In order to achieve these objectives, we will compare the fMRI maps of all three languages in left-handed and right-handed trilingual subjects and we will investigate whether the language activation on fMRI varies with the handedness of the subjects, with the age of language acquisition or with the language proficiency.

## Material and methods

### Subjects

The study protocol was reviewed and approved by the Institutional Review Board. 30 healthy trilingual adults were included in this prospective study. They were equally divided into two groups: a right-handed group and a left-handed group (Handedness was determined by the Edinburgh Handedness Inventory^36,37^). Ambidextrous subjects were excluded from the study. The two groups were matched for age and gender. Each group included eight women and seven men. The mean age was 27.2 years of age with a 2.8 SD in the right-handed group and 25.5 years of age with a 3.6 SD in the left-handed group. A medical history was recorded for each volunteer. Participants with neurologic, psychiatric, or other relevant medical disease were excluded from the study. All included subjects spoke English, French and Arabic. They were questioned about the time of acquisition of each language. For the purpose of the study, the native language was labeled L1, the second acquired language was labeled L2 and the third L3. L1 was acquired since birth. The mean age of acquisition of language for L2 and L3 was respectively 3 and 9 years of age. L3 was the English language for 97% of the subjects. L1 was the Arabic language for 70% of the population and the French language for 30% of the subjects. L2 was the French language for 70% of the subjects, Arabic for 26.7% of the subjects and English for the remaining 3.3%.

All subjects gave written informed consent to participate in the study and filled a questionnaire to objectively determine their language proficiency (using the Common European Framework of Reference for Languages CEFRL^38^). They were also asked to report their language habits including how many hours per day and in which context (social, professional) they use each language. Subjective proficiency was also assessed with the subjects ranking L1, L2 and L3 from the language they feel the most comfortable using (score of 1) to the language they feel the least comfortable using (score of 3).

17 of the 30 participants had a normal brain MRI. 13 of the 30 subjects had minor brain abnormalities: seven participants presented nonspecific signal abnormalities of the white matter, five had pineal cysts, and one subject demonstrated an arachnoid cyst. None of these abnormalities was significant enough to interfere with the results of the fMRI. Demographics of the population are summarized in Tables 1 and 2.

**Table 1:** Subjects demographics and language lateralization in the left-handed group.

Subject	Gender	Age	Handedness laterality score	L1	Age of acquisition	Subjective proficiency	Objective proficiency	Hours of use per day	Order of fMRI paradigm	Laterality Index	Lateralization	L2	Age of acquisition	Subjective proficiency	Objective proficiency	Hours of use per day	Order of fMRI paradigm	Laterality index	Lateralization	L3	Age of acquisition	Subjective proficiency	Objective proficiency	Hours of use per day	Order of fMRI paradigm	Laterality index	Lateralization
L1	M	32	− 88.2	A	0	2	C2	5.5	1	0.71	L	F	3	3	B2	0.5	3	0.71	L	E	10	1	C2	10	2	0.64	L
L2	F	23	− 78.9	A	0	1	C2	8	1	1	L	F	2	2	C2	7.5	3	1	L	E	8	3	C1	0.5	2	1	L
L3	F	28	− 100	A	0	3	C2	8	3	− 1	R	F	3	2	C2	4	2	− 0.45	R	E	10	1	C2	4	1	− 0.36	R
L4	F	21	− 100	A	0	1	C2	10	1	0.33	L	F	4	3	B2	1	2	0.2	B	E	12	2	C1	5	3	0.57	L
L5	M	27	− 100	F	0	2	C2	2	3	0.43	L	A	2	1	C2	13	2	0.47	L	E	7	3	C2	1	1	0.81	L
L6	F	24	− 100	A	0	1	C2	9	1	0.6	L	F	5	3	C1	1	2	0.37	L	E	8	2	C1	6	3	0.73	L
L7	F	29	− 88.2	F	0	1	C2	8	2	0.22	L	E	3	3	C2	4	3	0.1	B	A	8	2	C1	4	1	0.35	L
L8	M	23	− 100	F	0	1	C2	6.5	2	0.15	B	A	3	2	C2	6.5	2	0.52	L	E	9	3	C2	3	1	0.66	L
L9	M	24	− 100	A	0	1	C2	10.5	3	0.38	L	F	4	2	C2	4	2	0.31	L	E	7	3	C1	1.5	1	0.19	B
L10	M	27	− 100	A	0	1	C2	14	2	1	L	F	3	2	C2	1	3	0.88	L	E	11	3	C1	1	1	1	L
L11	F	23	− 100	A	0	1	C2	11	3	0.69	L	F	3	2	C2	4	1	0.33	L	E	7	3	B2	1	2	0.59	L
L12	F	26	− 100	F	0	1	C2	12	2	0.26	L	A	2	2	C1	3	2	0.34	L	E	10	3	B2	1	3	0.59	L
L13	M	18	− 88.2	F	0	1	C2	5	3	− 0.78	R	A	3	2	C2	10	2	− 0.67	R	E	6	3	B2	1	2	− 0.43	R
L14	M	29	− 75	A	0	1	C2	8	2	0.89	L	F	3	2	C2	5	1	1	L	E	11	3	C1	3	3	0.88	L
L15	M	28	− 77.8	A	0	1	C2	10	2	0.82	L	F	2	2	C1	4	1	0.84	L	E	8	3	B2	2	3	0.71	L

**Table 2:** Subjects Demographics and Language Lateralization in the right-handed group.

Subject	Gender	Age	Handedness laterality Score	L1	Age of acquisition	Subjective proficiency	Objective proficiency	Hours of Use per day	Order of fMRI paradigm	Laterality index	Lateralization	L2	Age of acquisition	Subjective proficiency	Objective proficiency	Hours of use per day	Order of FMRI paradigm	Laterality index	Lateralization	L3	Age of acquisition	Subjective proficiency	Objective proficiency	Hours of use per day	Order of FMRI paradigm	Laterality index	Lateralization
R1	F	36	100	F	0	1	C2	4	1	0.61	L	A	5	2	C2	11	2	0.72	L	E	11	3	C2	1	3	0.76	L
R2	F	27	78.9	F	0	1	C2	8	3	0.34	L	A	6	3	C1	5	1	0.56	L	E	7	2	C1	3	2	0.32	L
R3	F	25	100	A	0	1	C1	10.5	2	0.93	L	F	5	2	C1	5	3	0.82	L	E	13	3	B1	0.5	1	0.86	L
R4	M	27	100	A	0	1	C2	14	2	0.43	L	F	3	2	C1	1.5	1	0.4	L	E	4	3	C1	0.5	3	0.54	L
R5	M	25	100	A	0	1	C2	12	3	0.52	L	F	4	2	C1	3	1	0.55	L	E	8	3	C1	1	2	0.72	L
R6	M	27	100	A	0	1	C2	9.5	2	− 0.6	R	F	3	3	C2	2.5	3	− 0.67	R	E	9	2	C2	4	1	− 0.75	R
R7	F	27	100	A	0	1	C2	10	1	0.78	L	F	5	2	C2	5.5	2	0.83	L	E	7	3	C1	0.5	3	0.82	L
R8	M	29	100	A	0	1	C2	10	3	0.47	L	F	3	2	C1	4	1	0.59	L	E	12	3	B2	2	2	0.67	L
R9	M	26	84.6	A	0	1	C2	9	1	1	L	F	3	3	C2	4	2	1	L	E	8	2	C2	3	3	0.85	L
R10	F	28	90	A	0	1	C2	11	2	0.68	L	F	3	2	C1	4	1	0.5	L	E	8	3	B2	1	3	0.46	L
R11	F	28	100	F	0	1	C2	8	3	0.75	L	A	3	2	C2	5	1	0.59	L	E	9	3	C1	2	2	0.85	L
R12	M	25	90	A	0	1	C1	12	3	1	L	F	2	2	C1	6	1	1	L	E	9	3	C1	1	3	0.78	L
R13	M	25	100	F	0	2	C2	10	2	0.36	L	A	3	1	C2	4	3	1	L	E	9	3	C1	2	1	0.61	L
R14	F	25	100	A	0	1	C2	10	2	0.47	L	F	3	2	C2	5.5	3	0.52	L	E	9	3	C1	0.5	1	0.5	L
R15	M	29	85	A	0	3	C2	10	3	0.73	L	F	4	1	C2	4	1	0.53	L	E	10	2	C1	2	2	0.51	L

### Data acquisition

Language fMRI study was performed for every subject. Scanning was performed on a 3 T scanner (GE Healthcare, Milwaukee, Wisconsin) using an eight-channel head coil. We started the examination with an Axial FLAIR sequence (TR/TE = 10,000/140.2 ms, FOV = 220 × 220 mm, flip angle = 90, thickness = 4 mm) to rule out parenchymal abnormalities. Three-dimensional Axial T1-weighted images were acquired with a spoiled gradient-recalled echo sequence (TR/TE = 8.4/2.6 ms, FOV = 260 × 260 mm, flip angle = 15, matrix = 256 × 256 voxels, thickness = 1.2 mm). fMRI data were then acquired with a single shot gradient echo echo-planar imaging (EPI) sequence (repetition time/echo time (TR/TE) = 3,000/30 ms, field of view (FOV) = 260 × 260 mm, flip angle = 90°, matrix = 128 × 128 voxels, thickness = 4.5 mm, no skip, EPI voxel size = 1.875 × 1.875 × 4.5 mm).

### fMRI activation task

We used a visual responsive naming paradigm^39^ to activate Wernicke’s and Broca’s areas. The subjects underwent language fMRI with the same paradigm administered in French, English and Arabic. The participants were instructed to read and answer the questions silently, while using the same language the paradigm was administered in.

The order of administration of L1, L2 and L3 paradigms was random and independent of the age of acquisition and language proficiency of the subject. A block design was used in accordance with previous protocols of language system evaluation^40–42^. There was a sequence of 30 s of activation (five questions) followed by a rest period where the subject was asked to fixate on a cross-hair for 30 s. This was repeated five times (5 min in total). The blocks of activation (Epoch) were constructed similarly for each language and consisted of the same questions for each language. The order of the questions was however random within each block. The order of the blocks within each paradigm was also different to account for the effect of habituation.

A trained trilingual neuroradiologist performed the fMRI for all the participants and provided them with oral instruction before the fMRI. Subjects were monitored continuously while performing the fMRI. Subject participation was confirmed using real-time imaging software, which provided real-time display of functional results (BrainWave RT, GE Healthcare, Milwaukee, Wisconsin). BrainWave was used primarily for a quick look at task activation at the scanner console, to monitor patient's performance.

### fMRI data analysis

Post processing and analysis of the fMRI images were performed using SPM12 (https://www.fil.ion.ucl.ac.uk/spm/software/spm12/). The images of every subject were realigned using the mean image as a reference and normalized into the International Consortium for Brain Mapping (ICBM) space template for European brains. Smoothing was then performed with a Gaussian kernel of 8 mm full-width at half maximum, to improve signal to noise ratio.

Statistical fMRI analyses were performed at a single subject level and were followed by a group level analysis for the left-handed and the right-handed groups.

Statistical parametric maps were generated using a General Linear Model approach, and signal changes over time were correlated with a mathematical model of the hemodynamic response to neural activation. Rest and task conditions were compared using a t test at *p* < 0.001 (uncorrected). Functional activation maps were generated for each subject to allow for individual analysis.

### Language evaluation

The number of active clusters in the language areas and the number of active voxels (voxel size of 2 × 2 × 2 mm) in a cluster were reported by a neuroradiologist with 6 years of experience, blinded to the handedness of the subject, the age of acquisition and the language proficiency. Activation during task in comparison to rest was measured as number of activated voxels in a region of interest (ROI) over each of the main language areas in the bilateral hemispheres. The main language regions included the inferior frontal gyrus (IFG), the middle frontal gyrus (MFG), the superior frontal gyrus (SFG), the superior temporal gyrus (STG), the middle temporal gyrus (MTG), the supramarginal gyrus and the angular gyrus.

### Language dominance

The language laterality index (LI) was calculated for every subject for L1, L2 and L3 using the standard LI^43,44^ formula: LI = (*L − R*)/(*L* + *R*), where *L* and *R* are the numbers of voxels in the clusters of the main language areas in the left and right hemispheres, respectively. The LI ranged from − 1 (complete right dominance) to + 1 (complete left dominance). Right hemisphere language laterality was defined as − 1 ≤ LI <  − 0.2, bilaterality as − 0.2 ≤ LI ≤ 0.2, and left hemisphere language laterality as 0.2 < LI ≤ 1.

### Global activation of language

The global activation of language (GA) representing the total activation of the language areas in the left and the right hemispheres was calculated as GA = L + R.

### Statistical analysis

Statistical analysis was performed using SPSSv23. The correspondence between the LI in L1 and L2, L1 and L3, and L2 and L3 was evaluated in the right-handed and the left-handed groups. Correlations between the LI were determined with Pearson’s test. One-way ANOVAs examining the AoA of language effects on the GA was performed in each group. GA was also compared between the right-handed and the left-handed groups for L1, L2, L3 and for all languages using the Independent-Samples t-test.

Correlation between the order of the language paradigm administration during the fMRI, the subjective and objective language proficiency and the GA was performed using Spearman’s rank correlation.

Because our hypotheses concerned a priori differences between AoA of languages in each group, statistical tests between L1, L2 and L3 in the right-handed group and in the left-handed group were performed, with a Bonferroni correction (α = 0.012 [0.05 divided by 4 tests per group]).

### Group differences for L1, L2 and L3

The group maps for L1, L2 and L3 were created with SPM12 for the left-handed and the right-handed population using one-sample t-test. The results were corrected for multiple comparisons (Family Wise Error corrected) and thresholded at p < 0.05 with a minimum cluster size of ten voxels. The number of clusters and active voxels within clusters were compared between L1, L2, and L3 in the left-handed group and in the right-handed group.

### Ethical statement

The study has been approved by the Institutional Review Board and ethics committee and has therefore been performed in accordance with the ethical standards laid down in the 1964 Declaration of Helsinki and its later amendments. All subjects gave written informed consent in order to participate in the study.

## Results

### Group level results

**Figure 1:**
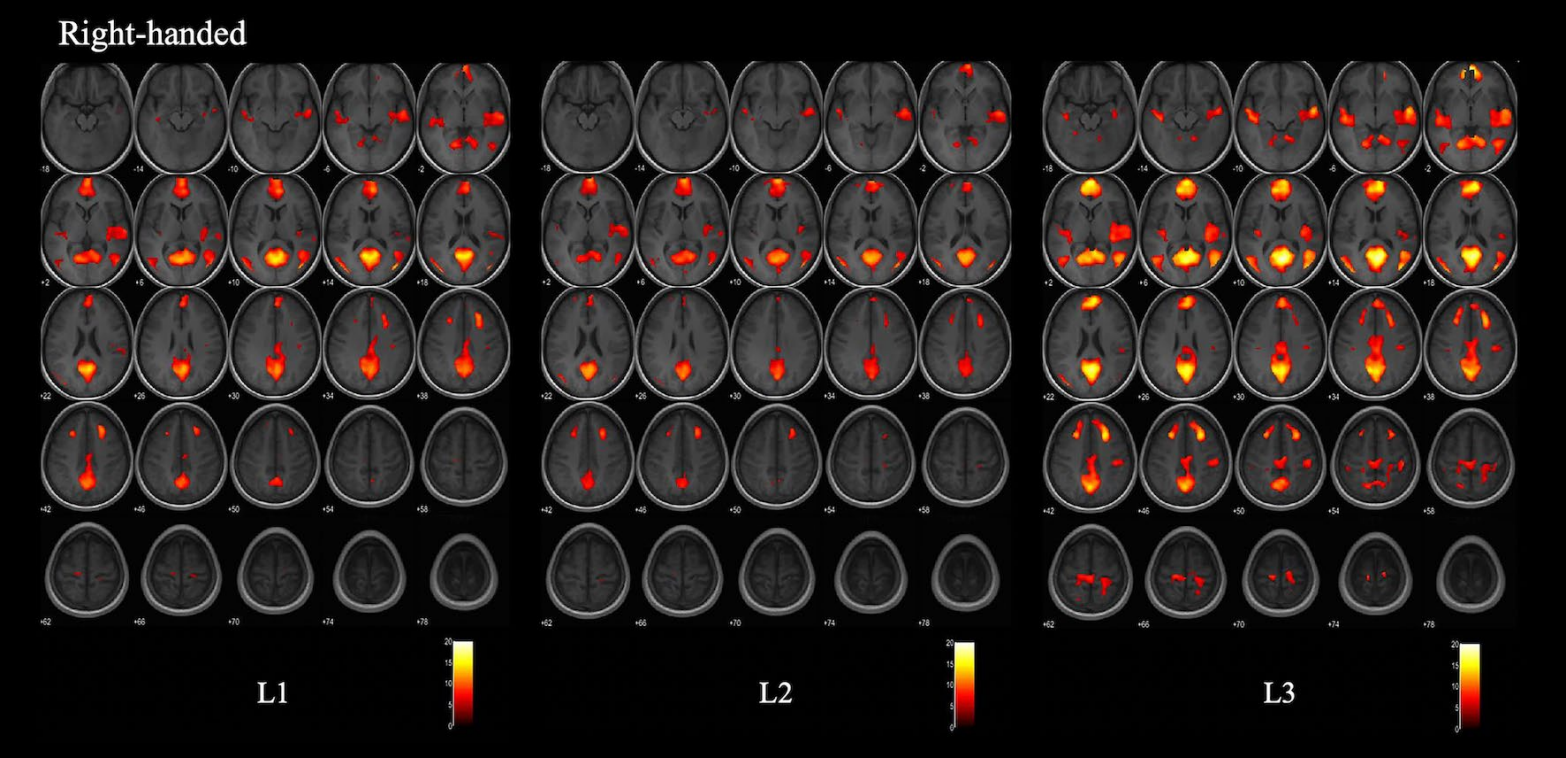
Group maps of L1, L2 and L3 for the right-handed subjects. Functional maps were obtained with a Family Wise Error correction, a threshold at p < 0.05 and the same T value range. They were overlaid on an average axial T1-weighted sequence of the brains of the 15 right-handed subjects. L1 and L2 present very similar activation maps. L3 presents larger areas of language activation, higher T values and more prominent activation in the accessory language areas in the bilateral superior gyri as well as the left post-central gyrus.

**Figure 2:**
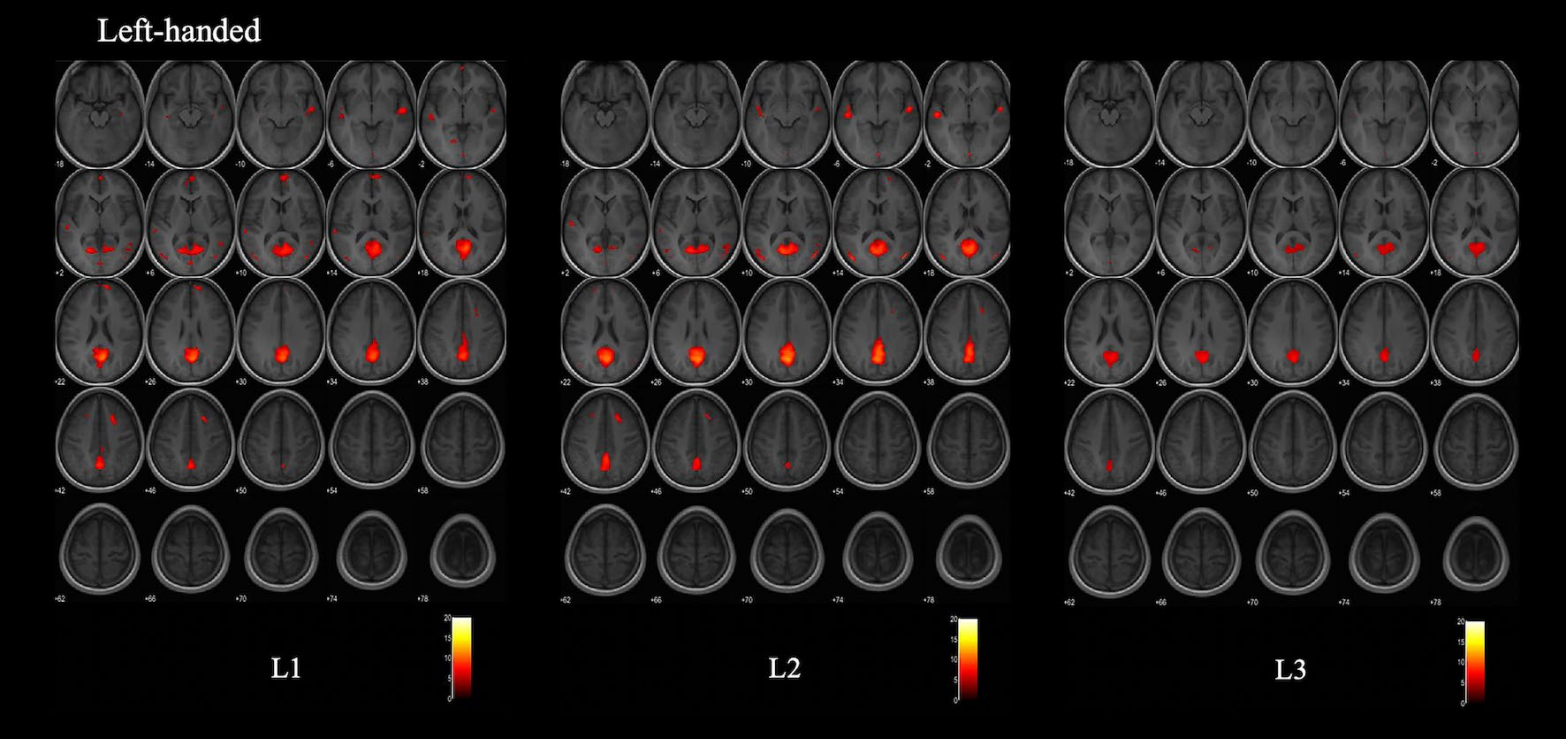
Group maps of L1, L2 and L3 for the left-handed subjects. Functional maps were obtained with a Family Wise Error correction, a threshold at p < 0.05 and the same T value range. They were overlaid on an average axial T1-weighted sequence of the brains of the 15 left-handed subjects. L1 and L2 present very similar activation maps. The L3 group map presents less activation than L1 and L2 and very small activation in the bilateral temporal lobes.

At a group level, there was left hemispheric and right hemispheric activation in both the left-handed and right-handed groups. There was an overlap of the main areas of language for all languages and especially for L1 and L2 that presented fairly comparable maps. However, the activation map of L3 was different than the activation map for L1 and L2 in both groups. The right-handed group activated a larger number of clusters and voxels with L3 compared to L2 and L1. This was most prominent in the accessory areas of language localized in the frontal lobes (Fig. 1 and Table 3). The left-handed group presented the least activation with L3 compared to the two other languages (Fig. 2 and Table 4). For each of the three languages (L1, L2 and L3), the right-handed group demonstrated larger clusters and voxels activation compared to the left-handed group.

**Table 3:** Activation in the right-handed group with a Family Wise Error applied at p < 0.05.

	L1						L2						L3					
	cluster Size	T score	Z score	Coordinates (mm)			cluster size	T score	Z score	Coordinates (mm)			cluster size	T score	Z score	Coordinates (mm)		
				x	y	z				x	y	z				x	y	z
**Frontal regions**																		
R sup frontal gyrus medial seg	2,835	11.77	inf	6	50	12	2,633	13.21	inf	2	64	8	6,184	16.18	inf	8	56	18
R sup frontal gyrus	766	11.54	inf	24	28	40												
R pre-central gyrus	114	5.38	5.35	20	− 26	66	45	5.1	5.07	− 6	− 26	56						
R post-central gyrus		4.91	4.89	24	− 30	52	96	5.44	5.41	22	− 30	54						
R middle frontal gyrus							711	10.29	inf	24	22	44						
L SMA	155	6.33	6.29	− 16	− 22	64	45	4.83	4.81	2	− 28	60						
L sup frontal gyrus	27	6.21	6.17	− 14	36	52	313	4.96	4.94	− 20	40	40						
L middle frontal gyrus	250	9.95	inf	− 24	22	42		8.39	inf	− 24	20	42	1,011	12.9	inf	− 24	22	46
L pre central gyrus							15	5.12	5.1	− 16	− 24	68						
L post central gyrus													120	6.4	6.35	− 44	− 34	56
R SMA							12	5	4.97	6	− 16	48						
**Temporal and parietal regions**																		
R superior temporal gyrus	2,415	7.09	7,03	54	− 26	0	1,600	9.7	inf	56	− 12	− 2						
R posterior insula		9.49	inf	62	− 16	0		6.73	6.67	40	− 14	10						
R middle temporal gyrus							936	7.32	7.25	46	− 56	14	1942	12.45	inf	46	− 58	10
R angular gyrus								7.19	7.12	56	− 58	14						
R Hippocampus																		
R supramarginal gyrus																		
L superior temporal gyrus	832	7.73	7.64	− 52	− 10	− 8	382	8.48	inf	− 52	− 8	− 8	1867	10.84	inf	− 48	− 18	− 10
L posterior insula		7.23	7.16	− 52	− 22	− 2												
L middle temporal gyrus							769	9.82	inf	− 54	− 68	12	1,395	11.28	inf	− 52	− 66	10
L angular gyrus													80	6.64	6.59	− 36	− 24	34
L fusiform gyrus	52	5.95	5.91	− 30	− 44	− 22							128	6.87	6.82	− 26	− 44	− 18
L precuneus	8,400	15.15	inf	− 6	− 58	16	5,632	12.56	inf	− 6	− 60	18	18,614	16.96	inf	− 6	− 58	16
R precuneus		14.73	inf	4	− 60	18		13.1	inf	2	− 64	16		19.43	inf	4	− 64	10
**Occipital regions**																		
R lingual gyrus																		
Rmiddle occipital gyrus	1,243	12.76	inf	42	− 78	12	936	11.42	inf	46	− 76	12	1942	13.65	inf	42	− 76	14
R inferior occipital gyrus		9.49	inf	50	− 68	4												
L middle occipital gyrus	880	11.31	inf	− 38	− 84	16	769	10.84	inf	− 46	− 76	16	1,395	12.31	inf	− 38	− 84	18
**Cerebellum**																		
R cerebellum exterior													57	5.39	5.36	26	− 44	− 22
R Thalamus													22	5.41	5.38	14	− 30	0

**Table 4:** Activation in the left-handed group with a Family Wise Error applied at p < 0.05.

	L1						L2						L3					
	cluster size	T score	Z score	Coordinates (mm)			cluster size	T score	Z score	Coordinates (mm)			cluster size	T score	Z score	Coordinates (mm)		
				x	y	z				x	y	z				x	y	z
**Frontal regions**																		
R sup frontal gyrus medial seg	706	6.64	6.59	8	64	12												
R sup frontal gyrus		7.72	7.63	14	60	24	83	6.46	6.42	20	60	16						
R pre-central gyrus																		
R post-central gyrus																		
R middle frontal gyrus							237	7.36	7.29	20	22	44						
L SMA																		
L sup frontal gyrus	38	5.84	5.8	− 22	26	42	51	6.21	6.16	− 20	56	24						
L middle frontal gyrus																		
L pre central gyrus																		
L post central gyrus																		
R SMA																		
**Temporal and parietal regions**																		
R superior temporal gyrus	369	7.6	7.52	52	− 10	− 6	204	8.07	inf	54	− 8	− 4	19	4.74	4.72	56	− 8	− 4
R posterior insula																		
R middle temporal gyrus	215	6.03	5.99	52	− 52	8	500	5.88	5.85	58	− 58	6						
R angular gyrus																		
R Hippocampus	369	6.16	6.12	38	− 16	− 18												
R supramarginal gyrus	13	4.92	4.9	64	− 28	22												
L superior temporal gyrus							439	8.58	inf	− 54	− 18	− 4	34	5.08	5.06	− 52	− 18	− 4
L posterior insula																		
L middle temporal gyrus	394	6.32	6.27	− 54	− 20	− 2	370	6.85	6.79	− 52	− 66	12						
L angular gyrus	37	6.45	6.4	− 60	− 58	24	31	5.55	5.52	− 60	− 58	24						
L fusiform gyrus																		
L precuneus	4,566	8.23	inf	− 18	− 64	4	4,801	9.59	inf	− 6	− 66	12	2,449	6.59	6.53	− 6	− 60	20
R precuneus		11.04	inf	6	− 62	22		11.77	inf	2	− 64	30		7.52	7.45	4	− 62	28
**Occipital regions**																		
R lingual gyrus																		
Rmiddle occipital gyrus	145	7.37	7.3	44	− 78	10							65	5.9	5.87	44	− 76	20
R inferior occipital gyrus																		
L Lingual gyrus							93	8.36	inf	2	− 86	− 4	55	5.79	5.76	0	− 86	0
L middle occipital gyrus	280	6.96	6.9	− 46	− 78	20	370	8.7	inf	− 42	− 78	14	73	5.91	5.87	− 50	− 74	16
**Cerebellum**																		
R cerebellum exterior																		
R Thalamus																		

### Single subject results

#### Atypical language lateralization

Atypical language lateralization was observed in 1 of the 15 right-handed subjects who presented right lateralization of language and in 6 of the 15 left-handed subjects, 2 of them presented right lateralization and the other 4 presented bilateral laterization of language.

#### Concordance of LI

**Figure 3:**
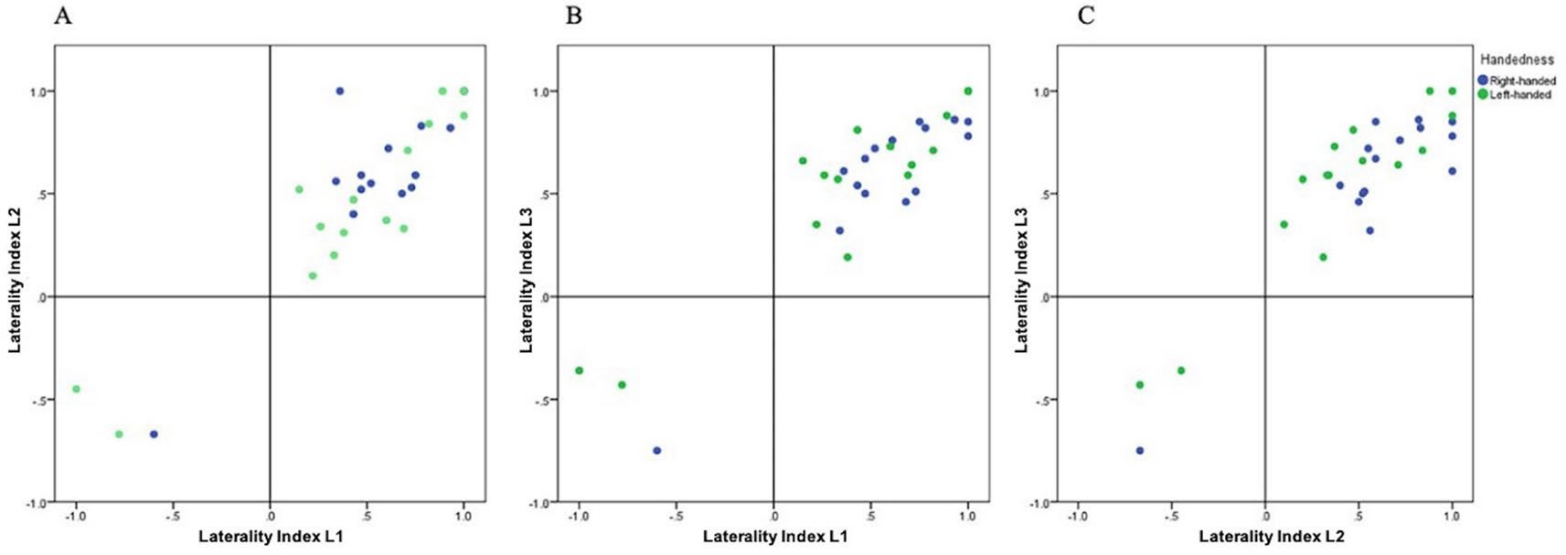
Correspondence between the LI in L1 and L2 (left), L1 and L3 (middle), L2 and L3 (right) in the right-handed subjects (blue dots) and the left-handed subjects (green dots). Pearson correlation showed strong correlation with p-values < 0.001 and coefficient factors ≥ 0.866.

There was good concordance of LI between L1, L2 and L3 (Fig. 3). Pearson correlation showed strong correlation between L1 and L2, L1 and L3, and L2 and L3, in both the right-handed and the left-handed groups, with p-values < 0.001 and coefficient factors ≥ 0.866*.*

Discordant lateralization of language was only observed in the four left-handed subjects who demonstrated a bilateral lateralization of language for one of the languages. They presented a left lateralization for the other two languages. The language that demonstrated a bilateral lateralization was the language with the least subjective proficiency in three of the four cases.

#### Global activation

Correlation between the global activation and the chronology of language acquisition in the right-handed group resulted in (ANOVA F (2, 42) = 3.268, p = 0.048) with the last acquired language L3 (after 6 years of age, mean age of acquisition: 9 years) inducing the highest degree of activation. This result was not significant after the Bonferroni correction. However, the group maps of activation for each language obtained in SPM12 with a Family Wise Error correction and p < 0.05 demonstrated a higher overall activation in the brain with L3 compared to L1 and L2, in the right-handed group only, confirming this result. There was no correlation in the left-handed group.

Spearman’s rank correlation analysis did not demonstrate correlation between the global activation and the order of the language paradigm administration during the fMRI. There was no correlation as well between the global activation and the subjective and objective language proficiency in right-handed and left-handed subjects.

There was a significant higher global activation for all languages (L1–L2–L3) in the right-handed group compared to the left-handed group (Independent-Samples t-Test 0.04). There was higher global activation for L1 and higher global activation for L3 between the right-handed and the left-handed groups (Independent-Samples t-Test 0.028 and 0.006).

## Discussion

One of the main objectives of this study was to evaluate language lateralization and organization in healthy left-handed multilingual subjects.

### Language lateralization

First of all, language lateralization in our left-handed trilingual population was consistent with the language lateralization of monolingual left-handed individuals described in the literature^34^: 22% of the left-handed subjects demonstrated an atypical lateralization of language with one of the three languages. Atypical lateralization included right lateralization and bilateral lateralization of language. On the other hand, in our right-handed population, 7% of the subjects presented an atypical lateralization of language which was only found to be a right lateralization of language. These findings match the previous results published by Knecht et al.^45^ who described right hemisphere dominance in 7.5% of their right-handed population and are compatible with the findings of Szaflarski et al.^34^ that showed that the incidence of atypical language lateralization in normal left-handed and ambidextrous subjects is higher than in normal right-handed subjects (22% vs 4–6%). This atypical lateralization of language in both the left- and right-handed populations confirms the need for language evaluation in a pre-operative setting in the right-handed and moreover in the left-handed patients.

Prior research has demonstrated that, typically, there is a good concordance between language LI values in bi- and multilinguals^5^. Centeno et al. reported that language lateralization can be reliably derived from fMRI tasks in a second language^4^. It is important to note that their population included only two left-handed patients out of 16. In our study, language lateralization was identical and the LI was concordant for all three languages in all the subjects in the right-handed group. This was irrespective of the objective and subjective language proficiency of the subjects and of the age of acquisition of the language. Thus, fMRI performed for a single language seems to reliably determine language lateralization in right-handed individuals.

However, in our left-handed group, lateralization of language varied for 4 of the 15 subjects. These participants demonstrated left lateralization for 2 of the languages, and bilateral lateralization for one of the languages. In most of the cases, the language presenting a bilateral lateralization was the language with the least subjective proficiency.

In left-handed individuals, fMRI would have to be performed for all three languages for evaluation of language lateralization, especially if the subject demonstrates left lateralization or bilateral activation for the first language tested. All the left-handed subjects showing right lateralization of language presented the same right-sided lateralization of language for all three languages.

### Language organization

The second objective of our study was to evaluate language organization and activation with L1, L2 and L3 and to determine whether the language activation on fMRI varies with the handedness of the subjects, with the AoA of language or with language proficiency. The group maps for L1, L2 and L3 in the right-handed and the left-handed group demonstrated a bilateral language activation with high activation in the right hemisphere as well as the left hemisphere. This finding confirms the theory of Hull and Vaid^17^ and the findings of Polczynska et al.^15^ of a more pronounced right hemisphere activation with early bilinguism, the mean AoA of L2 in our population was 3 years of age.

L1 and L2 which were both acquired before 6 years of age presented similar activation maps in both the right-handed and in the left-handed groups. This suggests that L1 and L2 might be both considered first languages^5^ in highly proficient early bilingual individuals. Note should be made that in our study, several factors between L1 and L2 were controlled. All our participants acquired L1 and L2 very early in life (before 6 years of age), they were born in L1 speaking families, learned L2 in preschool, followed a school and university education mainly in the L2 language, were highly proficient in both languages and continued communicating in both languages professionally and socially.

Compared to L1 and L2, L3 activation map was significantly different in both groups. In the right-handed group, the accessory language areas increased in number and size with L3 compared to L2 and L1. This might be explained by the fact that multilinguals have main areas that are common to all languages and additional language-specific sites^46^. Furthermore, it had been described that the activated volume increased for languages with poorer proficiency. Prior fMRI studies on multilinguals subjects showed that all languages activated overlapping areas, corresponding to the major language regions and that number of activated voxels inversely correlated with proficiency^12,16^. Even though our subjects also presented high proficiency in L3, in order to perform at the same proficiency level, the brain engages more accessory areas for later acquired languages^16^. This corroborates the findings of a previously published study by Perani et al. that more cerebral activation for the second language^10^ in a group of bilingual patients who had been exposed to L2 after the age of 3 and had the same level of proficiency in both languages.

The activation of the frontal lobes was more prominent and more extensive in L3 than in the two other languages. This is likely due to the late AoA of L3. Marian et al.^2^ described that a language with a late AoA may recruit the frontal lobe to a greater extent not because it is represented differently, but because it involves greater processing demands and more cognitive resources. It is difficult to determine if the larger activation noted with L3 in our right-handed group is due to a later AoA or to differences in proficiency between the languages as most of our subjects were post-doctoral fellows with high objective proficiency in L3.

In our right-handed population, the last language learned, with a mean age of acquisition of 9 years of age, induced the largest activation in the brain. Therefore, in healthy right-handed subjects, the language with the latest AoA will most likely result in the largest and more robust activation on fMRI.

However, it is interesting to note that this finding was not confirmed in the left-handed population. The group activation map of L3 for the left-handed subjects presented less activation in the brain compared to L1 and L2. It is however difficult to conclude that the language with a late AoA induces the least activation in the brain of left-handed subjects. As a matter of fact, the group map is a result that is obtained with SPM12 after correcting for multiple comparisons with voxelwise thresholding and Family Wise Error correction. This correction is an attempt at finding an appropriate balance between minimizing Type I error (false positives) and avoiding Type II error (omitting true effects)^47,48^. It can however result in omitting effects that are smaller and statistically weaker when dealing with complex neurologic process like processing of a second foreign language, especially when there is considerable individual differences as is seen in the left-handed population^34^. The small activation seen with L3 in the left-handed subjects might be due to statistical caveats rather than a true finding especially that it has not been described in the literature and that on a single level analysis the left-handed subjects showed as many areas of activation with L3, L2 and L1.

This study has limitations: first, our subjects can be considered a set of highly proficient individuals. While this would limit evaluation of the language organization in relation to proficiency, the homogeneity of our population permits to study the repercussion of an early vs late AoA of language. Second, different complexity level of each language was not controlled in the study design. A higher complexity processing of foreign languages is an intrinsic property of language processing in multilinguals. This factor will always be present when language lateralization and organization are evaluated in a routine pre-surgical setting. Third, differences in language organization in relation to the type of language were not evaluated in this study. Activation in relation to language similarity and to linguistic differences has been described in the literature with highly similar languages sharing neural representation while languages derived from different language families most of the time will not^18–20,49^. This theory was challenged by Polczynska et al.^15^ who operated on a quadrilingual woman and didn’t find that impairment across languages was related to language similarity. In our study, Arabic is a semitic language that derives from the Afro-Asiatic language family. It is written from right to left. French belongs to the Romance language family and German belongs to the Germanic language family and they both derive from the Indo-European language family. They are written from left to right. Thus, typologically, French and English are closer languages than French/English compared to the Arabic language. It’s interesting to note that, despite these differences, L1 and L2 maps in both groups were fairly similar, while L1 represented the Arabic language in 70% of the population, L2 the French language for 70% of the subjects, and L3 the English language for 96% of the subjects. Fourth, the activation in Broca’s area did not survive the Family Wise Error correction at p < 0.05 group analysis. This is probably due to the nature of the paradigm used. Further evaluation with other paradigms in the future would be helpful. Lastly, our study consisted of healthy volunteers only. Studies have shown that language laterality values can differ across languages in patients suffering from epilepsy or tumors^4,7^. Furthermore, L1 was found on cortical mapping to present more positive language sites than L2 and L3 in patient with high proficiency in foreign languages^13,25,26^. These observations demonstrate that it is recommended to map all languages spoken by surgical multilingual patients as some of them might present reorganization of one or several of their language networks due to the underlying brain pathology. It is interesting to note that very few left-handed subjects were included in these studies. In view of our findings, since left-handed subjects present more frequent atypical localization of language, possible discordant language lateralization across L1, L2 and L3 and less brain activation on fMRI compared to right-handed individuals, they should even more be tested for all languages in a preoperative setting.

## Conclusion

To summarize, the left-handed multilingual healthy subjects might present atypical lateralization of language and discordant language lateralization across the three languages. It’s therefore necessary to test all languages in left-handed multilingual subjects to determine language lateralization. The right-hemisphere presented prominent activation with the three languages, in both the left-handed and the right-handed groups, confirming the pronounced role of the right hemisphere in early bilinguism. L1 and L2, acquired before 6 years of age, presented similar activation maps and can both be considered first languages in highly proficient early bilingual individuals. L3, which was acquired after 6 years of age, presented the largest activation in the right-handed subjects only, confirming that in order to maintain a high-proficiency of language, more activation is needed in late multilingual right-handed individuals.
